# Deep-learning prediction of breast cancer hormone receptor status from CEM: a preliminary study

**DOI:** 10.1186/s41747-025-00653-3

**Published:** 2025-12-02

**Authors:** Alessandro Carriero, Marco Albera, Tomasina Meloni, Anna Rampi, Renzo Luciano Boldorini, Anna Colarieti

**Affiliations:** 1 Department of Translation Medicine, University of Eastern Piedmont (UPO), Novara, Italy; 2 SCDU Radiodiagnostica, Ospedale Maggiore Della Carità, Novara, Italy; 3 SCDU Anatomia Patologica, Ospedale Maggiore Della Carità, Novara, Italy

**Keywords:** Artificial intelligence, Breast neoplasms, Deep learning, Mammography, Precision medicine

## Abstract

**Background:**

Hormone receptor (HR) status guides breast cancer therapy. Deep learning (DL) applied to contrast-enhanced mammography (CEM) might offer a noninvasive means for HR status prediction, but class imbalance challenges model development and assessment. This preliminary study investigates CEM-based DL for HR status prediction, focusing on class imbalance handling.

**Materials and methods:**

In this retrospective study, CEM tumor crops from 105 patients with invasive breast cancer were used. Patients were randomized into training (*n* = 68), validation (*n* = 16), and independent test (*n* = 21) sets. A “Residual Network 18” (ResNet-18), pretrained on ImageNet, was fine-tuned using weighted cross-entropy loss and an Adam optimizer. Model selection used validation area under the precision-recall curve (AUPRC); output probabilities were calibrated via temperature scaling. Performance was reported with accuracy, area under the receiver operating characteristic curve (AUROC), and imbalance-aware metrics (balanced accuracy, Matthews correlation coefficient (MCC)) with 95% confidence intervals (1,000-iteration bootstrap). Results are presented for standard (0.5) and optimized (validation F1-score for HR-negative class) thresholds.

**Results:**

Validation AUPRC (model selection metric) was 0.640 (0.304–0.906). On the independent test set (optimized threshold 0.755), the model achieved 91.9% accuracy (86.5–97.3%), AUROC 0.808 (0.648–0.935), balanced accuracy 0.700 (0.550–0.853), and MCC 0.605 (0.296–0.818).

**Conclusion:**

A ResNet-18, utilizing patient-level data splitting and imbalance-aware fine-tuning, can capture CEM features for HR status, performing well despite significant class imbalance. Generalizability is limited by dataset characteristics and acquisition specifics, warranting further validation in larger, diverse cohorts to establish clinical applicability.

**Relevance statement:**

This work explores whether routinely acquired CEM images contain enough information for DL prediction of HR status. A ResNet-18 was trained with weighted loss and patient-level data splits; performance was quantified with imbalance-aware metrics to provide a realistic assessment in a highly skewed dataset, highlighting both the promise and current constraints of CEM-based molecular imaging.

**Key Points:**

- A ResNet-18, optimized for class imbalance through weighted training and with calibrated probabilities, predicted HR positivity on CEM with 91.9% accuracy and AUROC 0.81 in an independent test cohort using an F1-tuned threshold.
- Balanced accuracy (0.70) and MCC (0.60) demonstrate maintained discrimination despite an approximate 85% class imbalance (HR-positive cases).
- Patient-level splitting was employed to ensure robust evaluation.
- Limitations related to the dataset’s scope and specific imaging protocols may influence broader generalizability.

**Graphical Abstract:**

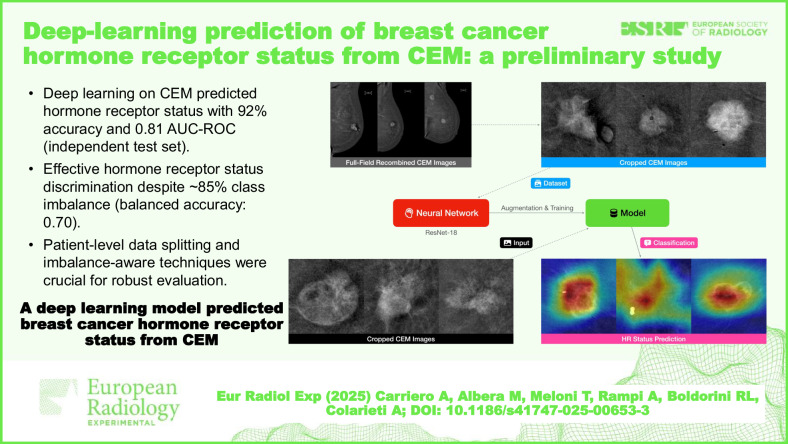

## Background

Breast cancer is the most frequently diagnosed malignancy worldwide, with about 2.3 million new cases and 670,000 deaths in 2022, according to the latest World Health Organization fact-sheet [1]. Because the disease is biologically heterogeneous, expression of the estrogen (ER) and progesterone (PR) receptors—summarized as hormone receptor (HR) status—remains a pivotal determinant of systemic-treatment strategy and prognosis [2]. HR status is routinely assessed on tissue obtained by core biopsy or surgery, but this invasive approach is time-consuming, prone to sampling error and limited in its ability to capture spatial heterogeneity [3, 4]. A rapid, image-based surrogate could therefore complement or, in selected scenarios, reduce the need for tissue sampling.

Contrast-enhanced mammography (CEM) merges dual-energy x-ray acquisition with an iodinated contrast bolus, highlighting lesion vascularity in a workflow that is faster and more widely available than magnetic resonance imaging (MRI) [5, 6]. Early clinical series reported higher sensitivity for lesion detection, particularly in dense breasts [7, 8], and recent reviews and surveys underline continued technical progress together with the emerging role of artificial intelligence tools and radiomics in breast imaging, including in CEM interpretation [9–11].

Concurrently, deep learning (DL)—especially convolutional neural networks—has transformed medical-image analysis. Surveys document expert-level performance across radiology domains [12]; ResNet and related architectures are now *de facto* baselines for image-classification tasks [13], and have already proven their ability to enhance breast cancer risk prediction in screening mammography [14]. Several groups have extended these methods to molecular profiling. Zeng et al [15] predicted ER, PR, and human epidermal growth factor receptor 2 (HER2) expression from digital mammograms with an area under the receiver operating characteristic curve (AUROC) up to 0.80. Zhang et al [16] also developed a multimodal DL model using mammography and ultrasound to predict molecular subtypes. Huang et al [17] and Ming et al [18] applied radiomics or multiscale convolutional neural networks on dynamic contrast-enhanced MRI to distinguish luminal from non-luminal cancers or to infer “prediction analysis of microarray 50” subtypes.

The application of artificial intelligence and radiomics to CEM is an emerging field of research. Fanizzi et al [19] proposed a radiomics-based malignancy detector; Qian et al [20] developed a multifeature fusion neural network for image classification, achieving high diagnostic performance, a finding discussed in an accompanying commentary on artificial intelligence in CEM [21]. Marino et al [22] applied texture analysis for molecular-subtype differentiation, while Dominique et al [23] trained a CheXNet-based DL model on cropped regions from CEM images to classify various histoprognostic factors, including ER and PR status, as well as triple-negative phenotype.

Collectively, these investigations have moved the field forward but share several limitations: they are predominantly single-center, rely on modest sample sizes, and often exhibit pronounced class imbalance. They also typically report only accuracy and AUROC without imbalance-aware metrics such as balanced accuracy or the Matthews correlation coefficient (MCC).

To provide a transparent benchmark that explicitly addresses class imbalance and ensures robust generalization estimates, we evaluated a ResNet-18 for HR prediction from CEM in a single-center preliminary study. Tumor regions were manually cropped to isolate lesion-specific signal while keeping preprocessing minimal. Model performance was reported with conventional metrics (accuracy, AUROC) and imbalance-aware measures (balanced accuracy, MCC) to reflect the ≈ 85% prevalence of HR-positive cases. Crucially, data splitting was performed at the patient level. Despite its limited scope, our work aims to establish a reproducible reference point for subsequent multicenter investigations.

## Materials and methods

### Study design and ethics

**Fig. 1:**
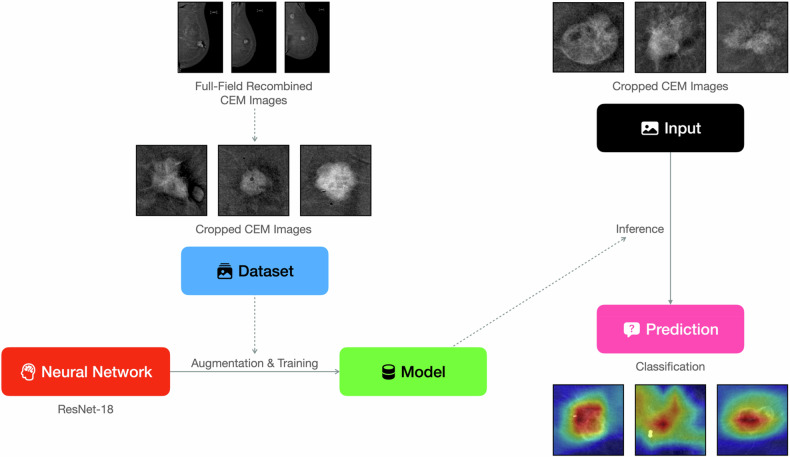
Schematic overview of the study pipeline: CEM acquisition, manual cropping, data augmentation, ResNet-18 training, and inference. CEM, Contrast-enhanced mammography; ResNet-18, Residual Network 18. See the following figures for details

This retrospective, single-center feasibility study was approved by the institutional ethics committee (see “Declarations”). The overall study pipeline is illustrated in Fig. 1. All 105 women analyzed had previously provided written consent for anonymized research use of their imaging and pathology data.

### Patient cohort

Women who underwent CEM between October 2020 and May 2022 for preoperative staging of biopsy-proven invasive breast cancer were screened. Departmental policy restricts CEM to patients aged ≥ 30 years, so younger women are absent. All tumors were clinical stage T1 or T2 at presentation. Contraindications to CEM (pregnancy, breast implants, impaired renal function, previous severe contrast reaction) preclude referral and are therefore not represented.

HR status was copied *verbatim* from each pathology report. A case was labeled HR-positive when either ER or PR was marked “positive” by the institutional pathology service. This reported status served as the reference standard for the study. HR-negative was considered the positive class (minority class, label ‘1’) for metrics like AUPRC and threshold optimization.

### CEM protocol

**Fig. 2:**
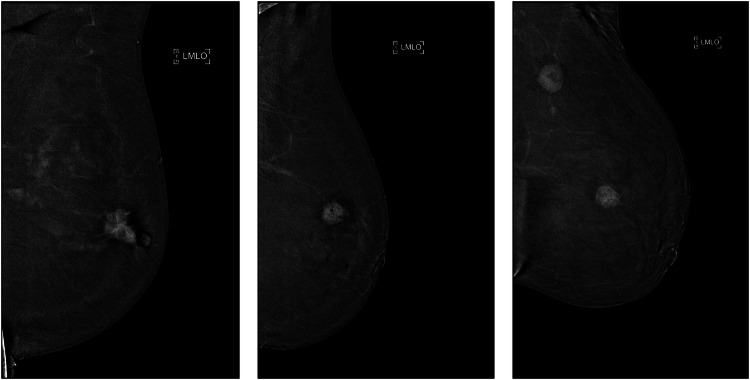
Representative recombined CEM mediolateral-oblique images illustrating the baseline appearance before cropping. CEM, Contrast-enhanced mammography

Imaging was performed on a Selenia Dimensions system (Hologic Inc.). Two min after intravenous administration of iodinated contrast (Iomeron 350, Bracco Imaging S.p.A.; 1.5 mL kg⁻¹; maximum 110 mL) at 2–3 mL s⁻¹, dual-energy two-dimensional craniocaudal and mediolateral-oblique views of the tumor-bearing breast(s) were acquired. Delayed images were also acquired 7 min after contrast injection. Only recombined (subtracted) images were analyzed (representative examples shown in Fig. 2). In patients with bilateral invasive cancers, both breasts were included; the laterality scanning order was not systematically enforced.

Images included in the analysis were deemed of clinically acceptable quality at the time of acquisition.

### Region-of-interest (ROI) definition and preprocessing

Enhancing lesions were localized on the recombined images and cropped manually with the workstation viewer (no external annotation software, no mirror-padding). Each rectangular crop covered the lesion and a small rim of surrounding tissue; multifocal tumors were cropped separately. The precise extent of the surrounding tissue and the approach in cases of very high or heterogeneous background parenchymal enhancement were based on the operator’s judgment to best encompass the visible lesion, which may introduce some variability.

**Fig. 3:**
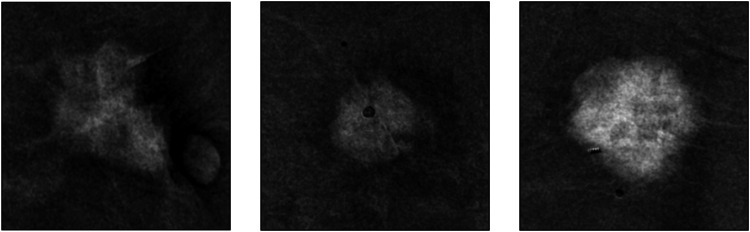
Representative manually cropped regions of interest used as input to the model

The resulting manually cropped ROIs (examples provided in Fig. 3) served as the input images for the model. These input images were converted to 3-channel grayscale. During training, crops were fed to a RandomResizedCrop((224,224)) layer, followed by RandomHorizontalFlip, RandomRotation(15), and ColorJitter(0.1,0.1). Validation and test images underwent Resize(256) and CenterCrop((224,224)). All images were normalized to the ImageNet mean and standard deviation.

### Dataset split

From the 105 patients (88 HR-positive, 17 HR-negative), 384 images were derived. Patients were randomly assigned, stratified by HR status, to a training set (68 patients: 57 HR-positive, 11 HR-negative; yielding 249 images: 213 HR-positive, 36 HR-negative), a validation set (16 patients: 13 HR-positive, 3 HR-negative; yielding 61 images: 52 HR-positive, 9 HR-negative) and an independent test set (21 patients: 18 HR-positive, 3 HR-negative; yielding 74 images: 64 HR-positive, 10 HR-negative). HR-positive cases comprised ≈ 85% of patients in every subset. This patient-level split ensures that images from the same patient do not appear in multiple subsets, mitigating information leakage. While this stratification aimed to balance the primary outcome, a detailed characterization of other clinical or imaging features (*e.g*., tumor size, grade, background enhancement) across the splits was beyond the scope of this preliminary study and represents a potential source of unassessed variability.

### Convolutional neural network architecture and training

A ResNet-18 pretrained on ImageNet served as the backbone. The choice of ResNet-18, a relatively less complex architecture, was deemed appropriate for the modest dataset size to mitigate overfitting while still benefiting from powerful pretrained features. The entire network was fine-tuned to adapt these features specifically to the CEM image domain and the HR status prediction task. The original fully connected layer was replaced by a single linear layer mapping ResNet-18’s 512 features to 2 (number of classes).

Training used the Adam optimizer with an initial learning rate of 1 × 10⁻⁵ and weight-decay of 5 × 10⁻⁴. The small initial learning rate was chosen to facilitate stable fine-tuning of the pretrained weights. A ReduceLROnPlateau scheduler reduced the learning rate by a factor of 0.1 after 7 epochs without improvement in validation AUPRC (HR-negative as positive class), allowing for adaptive adjustments during training. The loss function was weighted cross-entropy (weights 1.0 for HR-positive, 2.5 for HR-negative), in order to address the pronounced ≈ 85% class imbalance by compelling the model to pay greater attention to the minority HR-negative class. Mini-batch size was 4, and training ran for up to 30 epochs. Training and validation loss, as well as training and validation AUPRC, were recorded at each epoch. The network snapshot that achieved the highest validation AUPRC was retained. Following training, the model’s output probabilities were calibrated using temperature scaling, with the temperature parameter optimized on the validation set logits.

### Performance metrics and statistical analysis

Performance on the training, validation and independent test sets was summarized with: accuracy, specificity (recall for HR-positive), area under the precision-recall curve (AUPRC, HR-negative as positive class), balanced accuracy, MCC and AUROC. Precision, recall, and F1-score for both HR-positive and HR-negative classes were also calculated. Ninety-five percent confidence intervals (95% confidence intervals) were calculated with 1,000-iteration nonparametric bootstrap. An optimal classification threshold was determined on the validation set by maximizing the F1-score for the HR-negative class, and performance on all sets is reported using this optimal threshold alongside the standard 0.5 threshold. No hypothesis tests or *p*-values are reported, as only a single model was evaluated.

### Implementation and hardware

The pipeline was implemented in Python with PyTorch; full requirements and source code are available on GitHub (link in the Data-availability statement). Experiments ran on an M2 MacBook Air laptop (Apple Inc.) with 8 gigabytes of unified memory via the Metal Performance Shaders backend; the script automatically falls back to CUDA GPU or CPU if available. Temperature scaling was used for probability calibration. Gradient-weighted class activation mapping was used to generate activation heatmaps for model interpretability.

## Results

### Training behavior

**Fig. 4:**
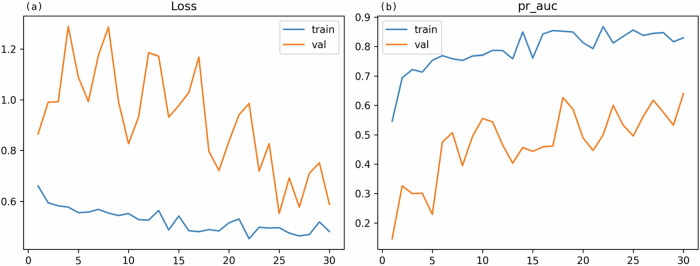
Training history over 30 epochs. **a** Weighted cross-entropy loss for training (blue line, train) and validation (orange line, val) sets. **b** Area under the precision-recall curve (AUPRC) for the hormone receptor-negative class for training (blue line, train) and validation (orange line, val) sets. The model from epoch 30, achieving the highest validation AUPRC of 0.6402, was selected

The training process over 30 epochs is illustrated in Fig. 4. Weighted cross-entropy loss for the training set generally decreased from an initial 0.6599 (epoch 1) to 0.4803 at epoch 30. Validation loss showed more fluctuation but ended at 0.5877 at epoch 30, having started at 0.8652 (epoch 1) and experiencing some peaks (*e.g*., 1.2886 at epoch 4) (Fig. 4a).

Training AUPRC (HR-negative as positive class) showed a general upward trend, increasing from 0.5459 at epoch 1 to 0.8296 by epoch 30 (Fig. 4b). The validation AUPRC, the primary criterion for model selection, fluctuated throughout training, achieving its maximum value of 0.6402 at epoch 30 (Fig. 4b). Consequently, the model checkpoint from epoch 30 was selected for final evaluation. After training, temperature scaling was applied to this model using the validation set, resulting in an optimal temperature of 1.386. This epoch-specific training AUPRC of 0.8296 reflects the metric as tracked during the training process for model selection purposes; the performance of this final selected and calibrated model on the full training set using the optimized threshold is reported in Table 1 as 0.9279. The ReduceLROnPlateau scheduler did not trigger a learning rate reduction during these 30 epochs.

**Table 1:** Performance metrics for the final ResNet-18 model on all data subsets, comparing a standard 0.5 classification threshold and the optimized threshold (0.755, F1-tuned on validation set for HR-negative class)

Metric	Training (0.5 threshold)	Training (optimized threshold 0.755)	Validation (0.5 threshold)	Validation (optimized threshold 0.755)	Test (0.5 threshold)	Test (optimized threshold 0.755)
Accuracy	0.7871 (0.7348–0.8354)	0.7068 (0.6466–0.7671)	0.6557 (0.5246–0.7705)	0.9180 (0.8361–0.9836)	0.6216 (0.5000–0.7297)	0.9189 (0.8649–0.9730)
Balanced accuracy	0.7876 (0.7328–0.8383)	0.7126 (0.6709–0.7566)	0.6603 (0.4681–0.8359)	0.7682 (0.6049–0.9403)	0.7391 (0.6026–0.8359)	0.7000 (0.5500–0.8530)
AUROC	0.8647 (0.8169–0.9080)	0.9033 (0.8634–0.9393)	0.7821 (0.5667–0.9709)	0.7821 (0.5667–0.9709)	0.8078 (0.6482–0.9351)	0.8078 (0.6482–0.9351)
AUPRC (HR-)	0.8838 (0.8370–0.9241)	0.9279 (0.8961–0.9544)	0.6402 (0.3044–0.9056)	0.6402 (0.3044–0.9056)	0.5817 (0.2754–0.8267)	0.5817 (0.2754–0.8267)
MCC	0.5755 (0.4687–0.6769)	0.5158 (0.4437–0.5922)	0.2327 (-0.0414–0.4824)	0.6387 (0.3199–0.8950)	0.3270 (0.1318–0.4908)	0.6047 (0.2961–0.8181)
Specificity/Recall (HR+)	0.7638 (0.6875–0.8368)	1.0000 (1.0000–1.0000)	0.6538 (0.5192–0.7843)	0.9808 (0.9361–1.0000)	0.5781 ( 0.4531–0.6970)	1.0000 (1.0000–1.0000)
Precision (HR+)	0.8083 (0.7391–0.8750)	0.6256 (0.5510–0.6927)	0.9189 (0.8095–1.0000)	0.9273 (0.8462–0.9831)	0.9737 (0.9091–1.0000)	0.9143 (0.8529–0.9718)
F1-score (HR+)	0.7854 (0.7249–0.8378)	0.7697 (0.7105–0.8185)	0.7640 (0.6500–0.8544)	0.9533 (0.9038–0.9910)	0.7255 (0.6170–0.8148)	0.9552 (0.9206–0.9857)
Recall (HR-)	0.8115 (0.7476–0.8781)	0.4252 (0.3419–0.5133)	0.6667 (0.3333–1.0000)	0.5556 (0.2222–0.9000)	0.9000 (0.6667–1.0000)	0.4000 (0.1000–0.7061)
Precision (HR-)	0.7674 (0.6923–0.8372)	1.0000 (1.0000–1.0000)	0.2500 (0.0952–0.4446)	0.8333 (0.5000–1.0000)	0.2500 (0.1176–0.4000)	1.0000 (1.0000–1.0000)
F1-score (HR-)	0.7888 (0.7325–0.8441)	0.5967 (0.5095–0.6784)	0.3636 (0.1538–0.5641)	0.6667 (0.3333–0.9091)	0.3913 (0.2051–0.5556)	0.5714 (0.1818–0.8277)

Values are point estimates (95% confidence intervals)

*AUPRC* Area under the precision-recall curve (for the HR- class), *AUROC* Area under the receiver operating characteristic curve, *MCC* Matthews correlation coefficient, *HR+* Hormone receptor positive, *HR-* Hormone receptor negative

### Final model performance

An optimal classification threshold of 0.755 was determined from the validation set based on maximizing the F1-score for the HR-negative class. Table 1 presents the discrimination results with 95% bootstrap confidence intervals for the training, validation, and independent test sets, comparing performance using a standard 0.5 threshold and this optimal threshold.

The modest gap between overall accuracy and balanced accuracy on the test set with the optimal threshold (91.9% *versus* 70.0%) highlights that while overall correct classification is high, performance on the minority class (HR-negative, recall 0.40 with optimal threshold, see full results for details) is more limited, as expected with high class imbalance, despite weighted loss and threshold optimization. Specificity (recall for HR-positive, the majority class) was 1.0000 on the test set with the optimal threshold.

**Fig. 5:**
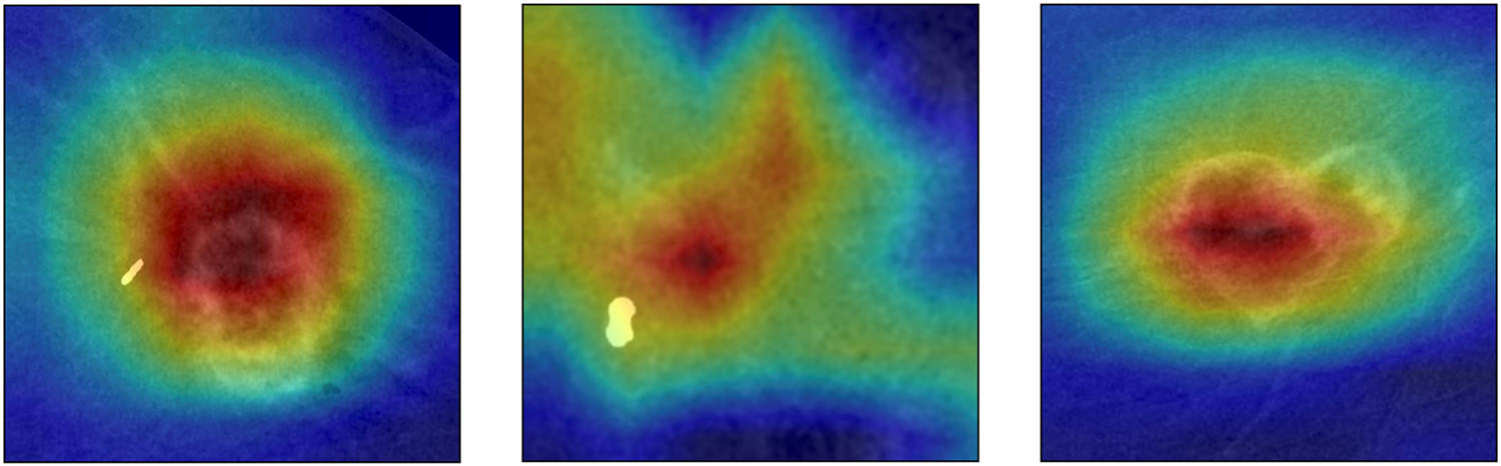
Grad-CAM heatmaps overlaid on cropped CEM images. Warm colors denote regions that contributed most to the hormone receptor status prediction. Grad-CAM, Gradient-weighted class activation mapping; CEM, Contrast-enhanced mammography

Figure 5 shows Gradient-weighted class activation mapping visualizations for representative cases, illustrating that network attention generally overlaps the enhancing tumor region.

Complete training (30 epochs) required approximately 10 min. Inference time per image was not formally measured.

## Discussion

In this retrospective study, we demonstrated that a ResNet-18, trained with patient-level data splits, weighted loss, and post hoc probability calibration, can predict HR status with 91.9% accuracy and an AUROC of 0.81 on an independent test set (using an F1-optimized threshold of 0.755). Reporting balanced accuracy (0.70) and MCC (0.60) alongside conventional metrics provides additional insight into model behavior under the pronounced class imbalance (≈ 85% HR-positive), and the use of patient-level splitting in this study ensures reliable estimates of model performance.

Placing these findings alongside existing literature requires careful consideration of methodological differences. Exploring CEM-based molecular cancer characterization, Marino et al [22] employed radiomics analysis and reported 78.4% accuracy for differentiating HR positive from HR negative cancers, evaluated using leave-one-out cross-validation. This contrasts with our DL approach and evaluation methodology, making direct metric comparison challenging. More recently, Dominique et al [23], in a large study of 389 patients, also used DL on cropped CEM images, reporting an area under the curve of 0.83–0.85 for ER prediction, while our study focused on the combined HR status (ER or PR positive) within a cohort of 105 patients. Looking at predictions from other modalities, Zeng et al [15] developed a DL model for standard, noncontrast mammography that achieved an area under the curve of 0.785 for ER prediction without manual segmentation of masses. Our use of CEM provides contrast-related information, and our model was trained on manually cropped tumor regions, highlighting different approaches to image input and feature extraction. These comparisons underscore that while the field is advancing, variations in patient cohorts, specific molecular targets (*e.g*., ER alone *versus* combined HR), imaging modalities or techniques (*e.g*., CEM *versus* standard mammography; DL *versus* radiomics), input data processing (*e.g*., cropped *versus* unsegmented), and evaluation methodologies make direct performance benchmarking complex.

Our study aimed to contribute a transparent reference point by employing a comprehensive methodology. The reported performance reflects the use of a ResNet-18 fine-tuned with weighted cross-entropy loss to address class imbalance, post hoc probability calibration using temperature scaling, and an F1-optimized classification threshold. Evaluation was performed using patient-level data splitting to ensure robust generalization estimates on an independent test set, with performance quantified using conventional and imbalance-aware metrics (balanced accuracy, MCC) to provide a realistic assessment, particularly given the pronounced class imbalance inherent in HR status prediction.

Several considerations temper the interpretation of our findings. First and foremost, the study draws on a small, single-center cohort (105 patients), which inherently limits external validity. Broader generalizability may also be affected by differences in CEM acquisition protocols (*e.g*., acquisition timing, injection rate, detector technology) that can vary between institutions. Moreover, for this investigation, no formal secondary review was undertaken to grade subtle artifacts, assess overall image quality, or examine laterality-related timing differences. Second, specific aspects of the dataset and its processing introduce potential variability: the manual ROI delineation, particularly in the presence of variable background enhancement, relied on operator judgment and could introduce variability not explicitly quantified in this study. Furthermore, the dataset was augmented by retaining late images (acquired 7 min after contrast injection in addition to standard 2-min images) to enlarge it; the influence of mixing these images is unknown but may have introduced unwanted variation. Additionally, lesion patches were resized with RandomResizedCrop during training, which can subtly stretch or compress the tumor, and the impact of this variable aspect ratio has not been quantified. Third, a limitation pertains to the reference standard, as HR status was copied *verbatim* from institutional pathology reports. While this ensures adherence to the clinical findings, it means the study relied on the reported classifications without independent verification of the underlying quantitative criteria. Finally, no parallel analysis of MRI or ultrasound was performed, so the relative diagnostic contribution of CEM cannot be inferred.

Despite these limitations, the study provides a transparent baseline for HR status prediction from CEM and underscores the value of imbalance-aware reporting and rigorous data splitting methodologies. Future research should incorporate multi-institution cohorts, harmonized acquisition protocols, automatic lesion localization, consensus pathology thresholds and direct modality comparisons to clarify the clinical role of CEM-based deep learning in molecular characterization.

In conclusion, a ResNet-18 convolutional neural network, fine-tuned with weighted loss and employing patient-level data splits, can extract informative features from contrast-enhanced mammography. In this study, following calibration of its output probabilities and application of an F1-optimized threshold, it predicted hormone receptor status with 91.9% accuracy and an AUROC of 0.81 on an independent test set. The accompanying balanced accuracy of 0.70 and MCC of 0.60 indicate that the model retains discriminative value despite a markedly skewed class distribution, although performance on the minority class remains a challenge. These results, achieved with minimal preprocessing and standard architecture, confirm the presence of a biologically relevant signal in CEM images. Extending this work to multicenter cohorts, harmonized CEM protocols, and automatic lesion localization will be the next essential steps toward assessing whether CEM-based deep learning can contribute meaningfully to noninvasive tumor characterization in clinical practice.

## Data Availability

The dataset used in this study is available from the corresponding author on reasonable request. Training code is available at https://github.com/radiodiagnostica/resnet-cem-histo.

## Abbreviations

AUPRC: Area under the precision-recall curve
AUROC: Area under the receiver operating characteristic curve
CEM: Contrast-enhanced mammography
DL: Deep learning
ER: Estrogen receptor
HR: Hormone receptor
MCC: Matthews correlation coefficient
MRI: Magnetic resonance imaging
PR: Progesterone receptor
ROI: Region of interest
